# Adjuvant interferon in the treatment of melanoma

**DOI:** 10.1038/sj.bjc.6690582

**Published:** 1999-08

**Authors:** M R Middleton

**Affiliations:** CRC Department of Medical Oncology, Christie Hospital, Manchester, M20 9BX, UK


British Journal of Cancer (1999) 80(11), 1679–1680
© 1999 Cancer Research Campaign
Article no. bjoc.1999.0582

Editorial

### Adjuvant interferon in the treatment of melanoma

**MR Middleton**

CRC Department of Medical Oncology, Christie Hospital, Manchester M20 9BX, UK

It is 3 years since the Food and Drug Administration approved high-dose interferon α-2b for the adjuvant treatment of resected melanoma at high risk of recurrence (American Joint Committee on Cancer stages IIB and III). The enthusiastic response to the results of the Eastern Co-operative Oncology Group (ECOG) 1684 trial in the USA has not been replicated in Europe. Although the study was the first to show a significant improvement in relapse-free and overall survival with an adjuvant treatment many oncologists have not been prepared to submit patients to such an arduous regimen without confirmatory trials. Ravaud et al (1999) confirm the toxicity reported by the ECOG 1684 investigators, with 50% and 47% of their patients experiencing a grade 3 or 4 toxicity during the induction and consolidation phases of high-dose interferon therapy respectively. Only two-thirds of the patients received at least 80% of the intended dose. They conclude that the 'Kirkwood' regimen cannot be accepted as standard therapy without confirmation from Intergroup study 1690 of the earlier results (Kirkwood et al, 1996).

The Intergroup trial, of high-dose interferon (as in ECOG 1684) versus low-dose interferon (3 Mu thrice weekly) versus observation, was recently unblinded and preliminary results were presented at the ASCO congress in Atlanta. Unfortunately they do little to help resolve the issues surrounding the adjuvant therapy of melanoma. Although the improvement in relapse-free survival with high-dose treatment seen in the ECOG 1684 trial is confirmed ($P = 0.05$; hazard ratio 1.28) there was no significant improvement in overall survival. A smaller benefit in relapse-free survival, which was not statistically significant, was seen in the low-dose arm ($P = 0.17$; hazard ratio 1.09). Pooling the results of the two studies shows an improvement in relapse-free survival ($P = 0.0021$; hazard ratio 1.34) but not in overall survival ($P = 0.21$; hazard ratio 1.13) for high-dose treatment.

This unexpected difference in the results of the two studies can be explained by an improvement in the outcome of those patients kept under observation. This was largely due to an increase in survival after relapse in patients under observation in the Intergroup trial (4.4 years vs 1.8 years for this group in ECOG 1684). This may be due to changes in staging and surgical techniques: for example, sentinel-node mapping has altered the stage III population, with many patients now exhibiting only microscopic disease. In the earlier trial, significant differences in overall survival were only seen amongst patients with clinically apparent lymphadenopathy prior to resection. There was only a trend towards improved survival in the (small) group of patients with clinically inapparent, but microscopically detected, lymph node involvement. It remains to be seen how interferon affects the outcome in these patients, if at all. An increase in the proportion of patients with only microscopic disease might explain the different conclusions, to date, of the ECOG and Intergroup trials. Alternatively, some observation arm patients crossed over to interferon at first relapse and this may have had an impact. Thirty eight of 121 observation patients who relapsed in the Intergroup Study were treated with interferon. These patients survived significantly longer than their counterparts who did not receive the drug (2.22 years vs 0.82 years; $P = 0.0021$). Although providing further evidence that interferon has activity against melanome, this raises the question of when it is best given to patients.

Given the Intergroup result, the use of low-dose adjuvant interferon may not be rewarding. Neither the Intergroup trial nor the earlier World Health Organization study 16 have shown any benefit in terms of either relapse-free or overall survival for this approach (Cascinu, 1995). Further data on this dose of interferon will be available when the United Kingdom Central Committee for Cancer Research 'AIMHIGH' trial reports.

There seems little doubt that high-dose interferon has an impact on melanoma, and can delay the time to relapse in high-risk melanoma patients. Both the ECOG and Intergroup studies indicate this, and there was a similar trend in a smaller North Central Cancer Treatment Group high dose study (Creagan et al, 1995). However, around four-fifths of patients treated with high-dose interferon experience moderate or severe toxicity. Since we do not know the mechanism by which interferon acts against melanoma, further work is required to establish which patients benefit from which dose and schedule of interferon. The final report of the Intergroup study will address this in part, since measures of effect on peripheral blood lymphocyte cytotoxicity form part of the study.

For now, is not possible to recommend high-dose interferon as standard adjuvant treatment for melanoma: the toxicity of the regimen demands a clear improvement in overall survival to justify its use. Patients with resected lymph node involvement should be advised of the results of the ECOG 1684 and Intergroup studies, so that they may come to an informed decision about adjuvant treatment of their melanoma. Meanwhile, future studies of adjuvant therapy in melanoma should include observation as their control arm.

*Received 11 February 1999*
*Accepted 22 February 1999*

### REFERENCES

Cascinelli N (1995) Evaluation of efficacy of adjuvant rlFNa-2a in melanoma patients with regional lymph node metastases. *Proc Am Soc Clin Oncol* **14**: A1296 (abstract)

Creagan ET, Dalton RJ, Ahmann DL, Jung SH, Morton RF, Langdom RM Jr, Kugler J and Rodrigue LJ (1995) Randomized, surgical adjuvant clinical trial of recombinant interferon α-2a in selected patients with malignant melanoma. *J Clin Oncol* **13**: 2776

Kirkwood JM, Strawderman MH, Ernstoff MS, Smith TJ, Bordern EC and Blum RH (1996) Interferon alfa-2b adjuvant therapy of high-risk resected cutaneous melanoma: the Eastern Co-operative Oncology Group trial EST 1684. *J Clin Oncol* **14**: 7–17

Ravaud A, Bedane C, Geoffrois L, Lesimple T and Delauney M (1999) Toxicity and feasibility of adjuvant high dose interferon alpha-2b in patients with melanoma in clinical oncological practice. *Br J Cancer* **80**: 1767–1769

*© 1999 Cancer Research Campaign*

